# EDITOR’S MESSAGE / MESSAGE DE LA RÉDACTION

**DOI:** 10.29173/jchla29918

**Published:** 2025-12-01

**Authors:** Ashley Farrell

**Affiliations:** JCHLA/JABSC Editor

Welcome to the December 2025 issue of the Journal of the Canadian Health Libraries Association.

As the incoming Editor, I would like to take a moment to recognize and thank the former Editor, Jessica McEwan, for all of her hard work and leadership on the Editorial Board over the last three years. I look forward to a great year ahead with Melissa Walter as Senior Editor, Katie Merriam as Junior Editor, Kate Merucci as Copy Editor, and Tyler Ostapyk as Production Editor. Thank you to the entire Editorial Board for all of their efforts and contributions in making this issue possible.

In this issue, we have a research article from Scott, Ayers, and Read that examines the data sharing practices of researchers funded by the Canadian Institutes of Health Research (CIHR).

We are also pleased to present the JCHLA/JABSC Student Paper prize-winning article by Anthonisen and Banks, which analyzes the overlap between bioinformatics LibGuides from Canadian universities.

In book reviews, Cunningham considers *Predatory publishing and global scholarly communications*. We finish the issue with three product reviews. Chaves reviews The Lens, Hilton considers DynaMed, and Young and Premji provide a joint review of OpenAlex.

We hope you enjoy the latest issue of JCHLA/JABSC!

**Ashley Farrell**

*JCHLA/JABSC Editor*

*Email:* editor@chla-absc.ca

Voici le numéro de décembre 2025 du Journal de l’Association des bibliothèques de la santé du Canada.

En tant que nouvelle rédactrice en chef, je tiens à remercier l'ancienne rédactrice en chef, Jessica McEwan, pour son travail dévoué et son leadership au sein du comité de rédaction au cours des trois dernières années. Je me prépare à vivre une année exceptionnelle avec Melissa Walter comme rédactrice en chef adjointe, Katie Merriam comme rédactrice junior, Kate Merucci comme éditrice de texte et Tyler Ostapyk comme éditeur de production. Je remercie l'ensemble du comité de rédaction pour ses efforts et sa contribution à la réalisation de ce numéro.

Dans ce numéro, nous présentons un article de recherche par Scott, Ayers et Read qui examine les pratiques de partage des données des chercheurs financés par les Instituts de recherche en santé du Canada (IRSC).

Nous sommes également heureux de présenter l'article gagnant du prix JCHLA/JABSC Student Paper, rédigé par Anthonisen et Banks, qui analyse les convergences entre les guides en bio-informatique des universités canadiennes.

Dans notre section de critiques de livres, Cunningham examine *Predatory publishing and global scholarly communications*. Nous terminons ce numéro par trois critiques de produits. Chaves critique The Lens, Hilton examine DynaMed et Young et Premji proposent une critique conjointe d'OpenAlex.

Nous espérons que vous apprécierez le dernier numéro de JCHLA/JABSC !

**Ashley Farrell**

*Directrice de la rédaction JABSC/JCHLA Courriel:* editor@chla-absc.ca

